# Application of Flame-Retardant Double-Layered Shell Microcapsules to Nonwoven Polyester

**DOI:** 10.3390/polym8070267

**Published:** 2016-07-22

**Authors:** Chloé Butstraen, Fabien Salaün, Eric Devaux, Stéphane Giraud, Philippe Vroman

**Affiliations:** 1 University Lille Nord de France, F-59000 Lille, France; chloe.butstraen@ensait.fr (C.B.); eric.devaux@ensait.fr (E.D.); stephane.giraud@ensait.fr (S.G.); philippe.vroman@ensait.fr (P.V.); 2 Ecole Nationale Supérieure des Arts et Industries Textiles (ENSAIT)/Laboratoire Génie des Matériaux Textiles (GEMTEX), 2, allée Louise et Victor Champier, F-59100 Roubaix, France

**Keywords:** microcapsules, nonwoven, resorcinol bis(diphenyl phosphate), flame retardant, padding

## Abstract

A microencapsulated flame retardant was used in order to produce a flame retardant nonwoven substrate. Melamine-formaldehyde polymer-shell microcapsules, containing Afflamit^®^ PLF 280 (resorcinol bis(diphenyl phosphate)) as the core substance, were coated by an outer thermoplastic wall (polystyrene (PS) or poly(methyl methacrylate)), before being applied to a core/sheet-type bi-component PET/co-PET spunbond nonwoven substrate using impregnation. The outer wall of the microcapsules was heated to the softening temperature of the thermoplastic shell in order to be bonded onto the textile fibres. The thermal stability of the microcapsules was examined using thermogravimetric analysis. The textile samples were observed with a scanning electron microscope, and the flame retardancy performance was evaluated using the NF P92-504 standard. The results show that the composition of the outer polymeric shell affected the thermal stability of the microcapsules, since the particles with a PS shell are more stable. Furthermore, the microcapsules were more located at the nonwoven surface without affecting the thickness of the samples. Based on the results of the NF P92-504 test, the flame spread rate was relatively low for all of the tested formulations. Only the formulation with a low content of PS was classified M2 while the others were M3.

## 1. Introduction

Thermal performances of protective textiles, and more specifically of flame-retarded protective textiles, have attracted more and more attention. They are mainly based on the thermophysical properties of the materials and the type of textile construction used to realize the textile substrate. Textile functionalization by material modification can be obtained in the following three main ways [1,2,3]: (i) incorporation of functional additives into the polymer melt or polymer solution before spinning [4,5]; (ii) chemical grafting of additives on the fibre surface with or without using linkers [6,7]; and (iii) formation of a coating onto the surface of fibre or fabric [8,9]. Each process has some advantages and drawbacks and the choice depends not only on the chemical nature of the substrate but also on the permanence of the treatment and the end use of the textile. Thus, spinning offers high permanence but is not practicable on natural fibre; chemical grafting requires the presence of reactive or functional chemical groups onto the surface of the substrate; and coating, the most universal method, is independent from the textile type; low amounts of additives can be used and this technique allows the combination of different functionalities [10]. Nonwovens are one of the fastest-growing segments of the textile industry. The development of flame-retardant (FR) nonwovens dates only from 1973, but over the two last decades it has gained more and more attention [11]. Polyester fibres are the main synthetic fibres used in the industrial manufacturing sector, and PET nonwovens are used for building and railway applications.

Over the two last decades, the main research of surface approaches to flame retardancy has focused on developing halogen-free flame retardants for textile fabrics. The latest techniques of flame retardant treatments include sol-gel, layer by layer (LbL), and plasma [12]. The LbL technique combines simplicity and thickness at the nanosclale level. Carosio et al. have shown that the LbL coatings deposited by spray have exhibited the most efficient protection for PET fabrics [13]. Nevertheless, despite the complete drip inhibition for PET samples, this treatment could not render the treated fabrics with a self-extinguishing property. Recently, a sol-gel finishing technique was used to confer flame retardant properties. Alongi et al. have investigated the effects of process parameters to render durability to flame retardant sol-gel finishes [14]. Whatever the treatment used, it shows poor durability, and other shortcomings are the preservation of the raw fabric characteristics, and also the commercial exploitation. Since the end of the 1990s, microencapsulation of flame retardant compounds in the textile area has been recognized to be an effective method not only to bring new functionalities to the substrate but also to overcome some problems of FR systems such as a weak water resistance, poor compatibility, toxicity, poor thermal degradation, etc. [15,16,17,18], or the modification of the textile properties such as softness and drape [19], to avoid undesirable properties, i.e., their chemical activity, volatility, or migration to the polymer surface. Furthermore, the encapsulation step leads to an increase of the heat transfer area, to a decrease of the reactivity of the core materials to reduce the interference with other material parameters, and to enhance the low heat conductivity and to make the manipulation of FR easier [20]. The choice of the polymer for the formation of the shell during the microencapsulation process must take into account the considered application and the required material processes. In the textile field, the polymers used should have good thermo-mechanical properties to resist to the thermal and mechanical demands during the implementation processes. Thus, the use of these particles requires a high thermal stability of them, mainly influenced by the shell and the core materials composition [21]. Microencapsulation of FR compounds is mainly limited to phosphate derivatives such as ammonium polyphosphate [22,23,24,25,26], di-ammonium hydrogen phosphate [27], or red phosphorus [28], which are moisture or water-sensitive materials.

Microcapsules are usually applied to a nonwoven substrate by conventional finishing processes, which require the use of an excess polymeric binder to fix the microcapsules to the fibres [29]. The main drawback of these methods is the modification of the physical properties of the textile such as the drape, the air permeability and the thickness, which are drastically reduced, etc. [30,31]. Over the last few decades, modification of the shell properties to fulfill practical requirements has attracted increasing interest, since it is possible to improve the thermal stability, conductivity, mechanical strength or modify the wettability of the shell. Amongst the modifications, the formation of microcapsules with a double-layered shell, in which the outer one is made of a thermoplastic polymer such as polystyrene [32], or polyethylene [33] can be bonded directly onto the textile substrate with a thermal treatment to the softening temperature or up to the melting temperature of the polymeric shell. The outer shells are made either during the microencapsulation process or after it by dispersion of the synthetized capsules in a medium containing the monomer, to induce suspension polymerization.

Aryl phosphate derivatives, such as bisphenol-A bis(diphenyl phosphate) (BDP) and resorcinol bis(diphenyl phosphate) (RDP), are well known flame retardants and have found various applications due to their good thermal stability, high efficiency, and low volatility [34,35]. They can act in the gaseous and condensed phases during the material burning process. In a non-charring thermoplastic polymer, they may be used with highly-charring co-additives, such as a phenolic resin in poly(butylene terephtalate) [36], or also combined with magnesium hydroxide in polyamide 6 [37], or encapsulated in a melamine-formaldehyde resin (MF) for a polypropylene application [38] to increase their efficiency. Even if BDP may be microencapsulated by in situ polymerization or sol-gel methods [39,40], its relatively high viscosity (13,000 mPa·s at 25 °C) and heat distortion temperature lead to some problems in processing. Thus, in this study, RDP was selected as FR due to its low viscosity (600 mPa·s) and a higher phosphorus content (10.8) compared to BDP. The aim of the work reported in this paper was to synthetize suitable microencapsulated FR to be incorporated into a PET/co-PET nonwoven without modifying the intrinsic properties of the textile material. The influence of the process formulation on the microcapsule formation was evaluated. The morphologies of the microcapsules were studied using optical and scanning microscopy (SEM). The structure of the double-layered shell polymer was analysed by FT-IR spectroscopy, and the thermal stability was evaluated using thermogravimetric analysis (TGA). This was done to provide baseline data to permit a comparative determination of the influence of these particles on the nonwoven after impregnation bonding. The physical properties of the modified nonwoven samples were controlled, and the fire properties were characterized using the NF P92-504 test. A part of this study has been presented during the Cost Action MP1105 Final Conference “Innovation in flame retardancy of textiles and related materials” [41].

## 2. Materials and Methods

### 2.1. Materials

MF prepolymer resin was used as the shell-forming and protector colloid (2-Acrylamido-2-methyl-1-propanesulfonic acid, Aldrich, France) employed as emulsifier were kindly supplied by Robert Blondel Cosmétiques (Malaunay, France). Afflamit^®^ PLF 280 (RDP) obtained from Thor (Salaise sur Sanne, France) was used as core materials. Formic acid, as pH control, glycidyl methacrylate (GMA) (as coupling agent), methyl methacrylate and styrene (as monomers for the outer shell) and benzoyl peroxide (BPO) (as initiator) were purchased from Sigma Aldrich (Saint-Quentin Fallavier, France).

A core-sheath PET/co-PET bi-component (85%/15%) spunbond nonwoven fabric with a weight of 262 g/m^2^, realized at CENT (Tourcoing, France), was used as the textile substrate. The sheath-core bi-component low-melt fibre, containing 85% PET and 15% of low-melt co-PET, was used for creating bonded contacts between fibres. The fineness of the two fibres is 6.7 and 2.2 dTex, for a length of 58 and 31 mm and a mean diameter of 24.9 and 14.2 mm, respectively.

### 2.2. Preparation of the Double-Layered Shell Microcapsules

**Figure 1:**
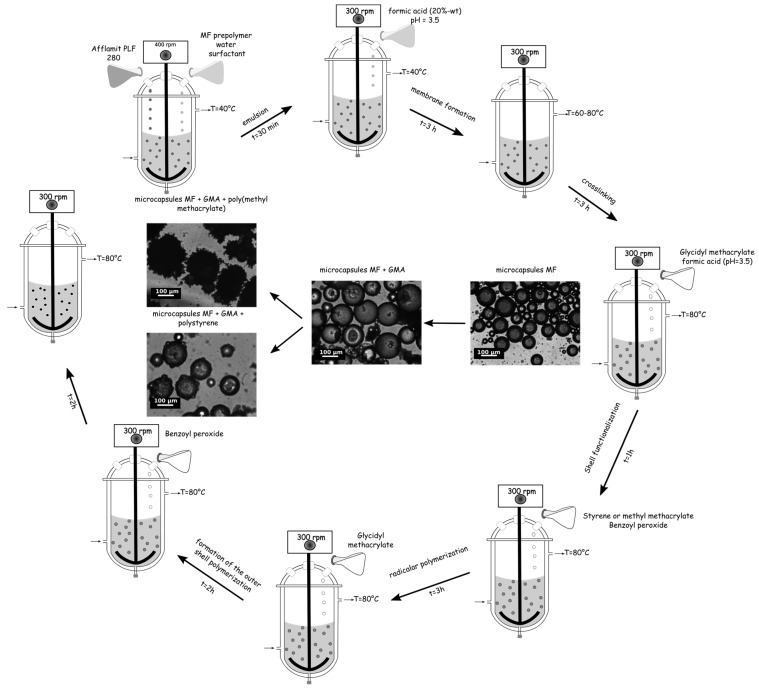
Schematic representation of the microencapsulation process.

The microencapsulation of RDP was carried out in a 500 mL three neck round-bottomed vessel equipped with a mechanical stirrer via an in situ polymerization based on our previous studies [27,42] followed by a suspension polymerization, according to the following method. The typical procedure for the preparation of these microcapsules was divided in two consecutive steps, i.e., (i) encapsulation of RDP by an amino shell; and (ii) entrapment of these particles by a polymer either PS or PMMA using a coupling agent (Figure 1).

#### 2.2.1. Preparation of MF Microcapsules

Twenty grams of RDP were emulsified in 20 mL of water containing 20 wt % of colloid protector and 5.4 g of MF prepolymer at 400 rpm with a mechanical stirrer for 30 min, until the mean diameter and size distribution reached the desired values. In this study, the size distribution should range from 10 to 100 µm, with less than 10% under 10 µm. After 30 min, the pH of the solution was adjusted to 3.5 with formic acid solution (20.0 wt %), and to initiate the polycondensation of methylol melamine with the formation of –O– or –CH_2_– bridge bond, the temperature of the solution was slowly increased to 60 °C, and the stirring rate was reduced to 300 rpm. After 3 h, to insure a completed crosslinking, the solution was heated to 80 °C for 3 h.

#### 2.2.2. Formation of the Outer Thermoplastic Shell

**Figure 2:**
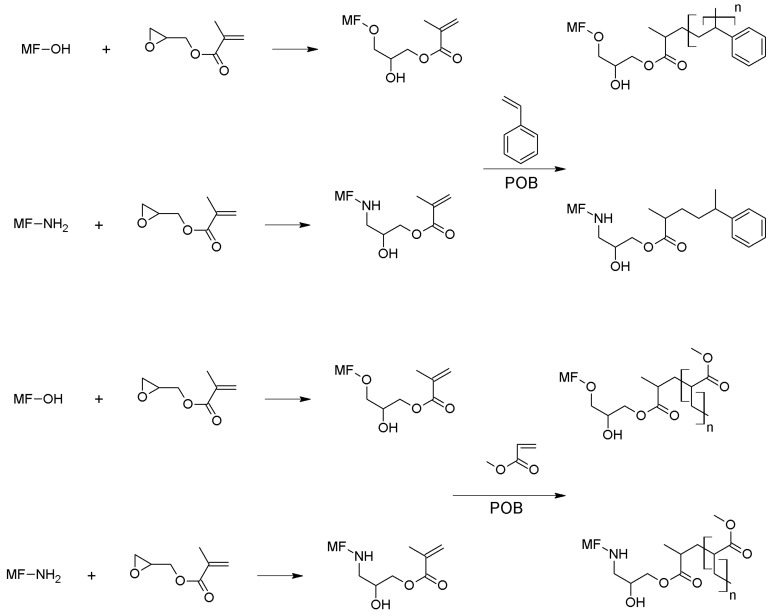
Proposed reaction between MF microcapsules, GMA, and styrene or methyl methacrylate.

One millilitre of glycidyl methacrylate was added dropwise to the above-mentioned microcapsule solution at pH = 3.5 at 80 °C under an inert atmosphere at 300 rpm. After one hour, 5, 10, or 20 g of monomer solution (methyl methacrylate or styrene) with benzoyl peroxide was slowly poured in the vessel to initiate the suspension polymerization for 3 h. To complete the formation of the outer shell, 1 mL of GMA at pH 3.5 with formic acid solution was added, and after two hours of continuous agitation, a small amount of BPO was introduced into the vessel (Figure 2). The pH of the solution was maintained at 3.5, and finally after two hours the regulation temperature and the stirrer were switched off. Once cooled to room temperature, the suspension of microcapsules was collected. The microcapsules were recovered by filtration, washed twice with ethanol and distilled water, and dried at room temperature overnight. The formulations and sample labels are listed in Table 1.

### 2.3. Preparation of Flame Retardant Nonwoven

A pad-dry-cure process was used to functionalize nonwoven samples with microcapsules, in 220 g/L, using a laboratory-scaled padder (Werner Mathis AG, Oberhasli, Switzerland). The pressure was set to 1 bar, and the rotation speed to 2.5 m/min. The microcapsules solution was initially prepared by dispersion of the particles in water. After impregnation, the samples were dried at 100 °C for 6 min and cured at 130 °C for 3 min for the PS-based samples, and 160 °C for 9 min for the PMMA samples, using a hot air dryer (Werner Mathis AG).

### 2.4. Characterizations

#### 2.4.1. Morphology of the Microcapsules and Functionalized Nonwovens

The microscopic aspects of the microcapsules and functionalized textile substrates were observed by both optical microscopy (Axiolab Zeiss, Carl Zeiss, Jena, Germany) equipped with a uEYE camera (IDS, Obersulm, Germany) and scanning electron microscopy (Philips XL30 ESEM/EDAX-SAPPHIRE, FEI, Eindhoven, The Netherlands).

#### 2.4.2. Infrared Spectroscopy

The structure of the shell polymer was analysed by FT-IR spectra. Samples were ground and mixed with KBr to make pellets. FTIR spectra in the absorbance mode were recorded using a Nicolet Nexus (Thermo Fisher Scientific, Villebon sur Yvette, France), connected to a PC, in which the number of scans was 128 and the resolution was 4 cm^−1^.

#### 2.4.3. Thermogravimetric Analysis of the Microcapsules

The thermogravimetric analysis (TGA) was carried out on a TA 2050 instrument (TA Instruments, Guyancourt, France) under a nitrogen atmosphere at a purge rate of 50 mL·min^−1^. For each experiment, a sample of approximately 10 mg was used. A heating rate of 10 °C·min^−1^ was applied, and the temperature was raised from 20 to 600 °C.

#### 2.4.4. Air Permeability & Thickness

The air permeability of nonwoven fabrics was determined by the rate of the flow of air passing perpendicularly through a given area of fabric by measuring at a given pressure its difference across the fabric test area over a given time period. Transverse air permeability was measured with FX3300 (Texttest AG, Schwerzenbach, Switzerland) with a pressure applied of 196 Pa, according to ISO 9237. The thickness of the materials was determined according to ISO 5084.

#### 2.4.5. Flame Retardant Properties

A rate of spread of flame test (NF P 92-504) was used as a guidance test in this work to evaluate the flame retardant properties of the functionalized nonwoven fabrics. This method is applied to fabrics that show an unusual behaviour during the electrical burner test. After burning, non-propagation of the flame and burning or not-burning droplets are observed. The sample (460 mm × 230 mm) is placed horizontally (i.e., the level of the short side, vertically, and the level of the long side, horizontally) and, counting from the free end, marks are placed at 50 mm and 300 mm, respectively. To test, the free end of the fabric is put in the flame of the gas burner, and the test sample is then put out. The fire burning time from the free end is measured 10 times, each for five seconds in length. The test sample flame spread rate of fire is the length of time imposed for 30 s. Determination of flame propagation is based on the time t between the two marker lines. Using the formula *V* = 250/t, the flame propagation rate is calculated and, thus, the material classification is established.

## 3. Results

### 3.1. Structures and Morphologies of the Microcapsules

**Figure 3:**
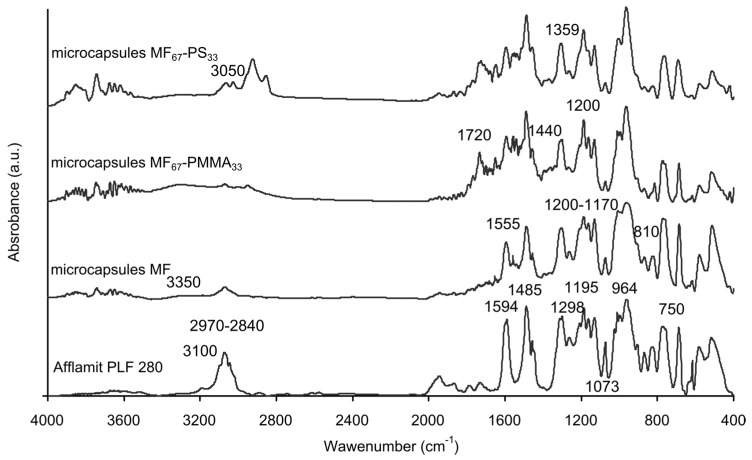
FTIR spectra of Afflamit PLF 280, MF microcapsules, MF_67_-PMMA_33_ microcapsules, and MF_67_-PS_33_ microcapsules.

The spectrum of Afflamit PLF 280, selected as the core solution, shows the characteristic IR peak of the phosphorus derivative compound. The peaks at 1594, 1485, and 840 cm^−1^ are assigned, respectively, to C=C stretching, and C–H bending in aromatic groups. The absorption at 1298 cm^−1^ can be ascribed to the P=O stretching in pentavalent phosphorus compounds. In addition, the absorption at 1195, 1073, and 964 cm^−1^ are related to Aro–O, P–O–(Ph) and P=O stretchings in the pentavalent phenyl phosphate, respectively [43]. Concerning the MF microcapsules, characteristic bands responsible for hydroxyl, imino, and amino stretching are observed around 3350 cm^−1^. Alkyl C–H are found between 2970 and 2840 cm^−1^, whereas CN stretching in the triazine ring and bending are present around 1555 and 810 cm^−1^. Furthermore, C–N vibration appeared between 1200 and 1170 cm^−1^ [41]. The presence of all of the characteristic bands for RDP can be clearly distinguished in the spectra of these microcapsules. Thus, the core solution has been successfully encapsulated by melamine–formaldehyde resin. The characteristic peaks of PMMA were observed at 1720, 1440, and 1200 cm^−1^, which can be assigned to C=O stretching, C–H bending, and C–O stretching vibrations of the ester group in Figure 3. For polystyrene outer shell microcapsules, the absorption peaks at 3050–3020 cm^−1^ are associated with the aromatic C–H stretching vibration, the absorption peak at 2930 cm^−1^ with the aliphatic C–H stretching vibration, the peaks at 1600 and 1495 cm^−1^ with the benzene ring C=C stretching vibration, and the peaks at 750–700 cm^−1^ with the benzene ring deformation vibration. Furthermore, these two latest spectra have also the characteristic bands absorption of the MF microcapsules, which denotes the presence of an inner MF shell and RDP core solution in these microcapsules (Table 2).

**Figure 4:**
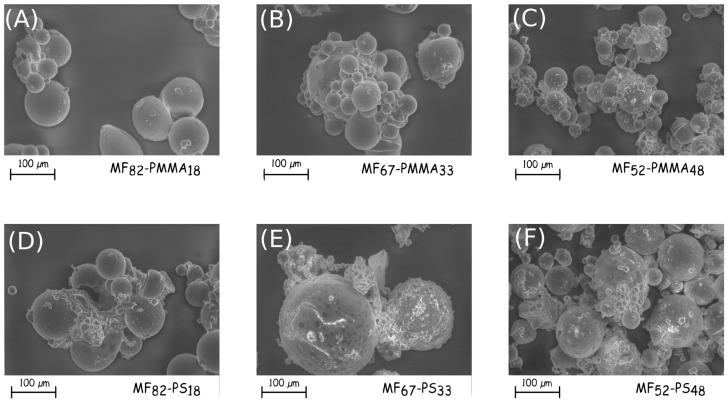
SEM micrographs of the synthesised double-layered shell microcapsules ((**A**) MF_68_-PMMA_18_; (**B**) MF_67_-PMMA_33_, (**C**) MF_52_-PMMA_48_, (**D**) MF_82_-PS_18_, (**E**) MF_67_-PS_33_ and (**F**) MF_52_-PS_48_).

In Figure 1, the modification of the microcapsules morphologies during the microencapsulation process can be noticed. After the MF shell formation, the particles having a size distribution from 10 to 100 µm seems to be relatively spherical with a smooth surface, the addition of GMA does not modify these characteristics. Furthermore, no destruction of the capsule walls due to mechanical stirring is perceivable. The presence of monomers, which allows the formation of the outer shell changes the morphologies. Indeed for the PS-based microcapsules, aggregation of particles appears and tends to decrease with the increase of monomer (Figure 4D–F). Additionally, tiny particles with a mean diameter less than 10 µm are deposited onto the surface, undoubtedly due to the post-treatment, and especially the washing and drying steps. The surface of these microcapsules is relatively smooth. The formation of the PMMA membrane leads to the aggregation of the microcapsules (Figure 4A–C). The optical observation shows also that the surface has a sea urchin-like structure. During the polymerization, the macromolecular chains formation occurs from the coupling agent and develops itself in the continuous phase. After washing and drying, the polymer collapses onto the surface to form a uniform coating, which also induces the aggregation. Furthermore, the roughness of the shell increases with increasing the PMMA amount. The final morphology and the presence of agglomerate are mainly related to the choice of the outer thermoplastic shell.

### 3.2. Thermal Stability of the Microcapsules

**Figure 5:**
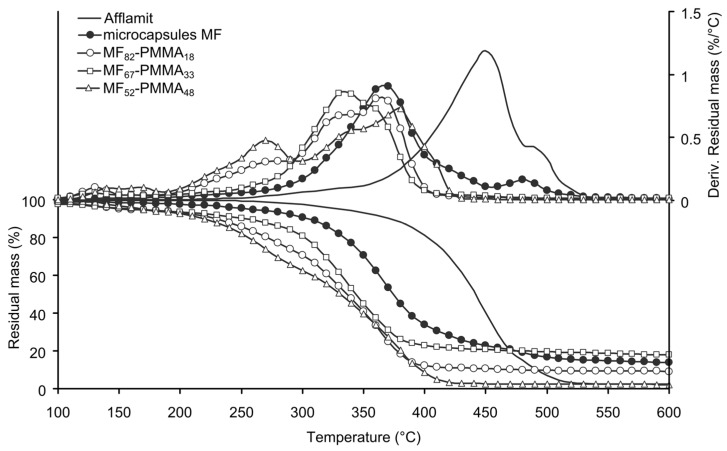
TG and DTG of RDP, MF microcapsules, and PMMA-based microcapsules (N_2_, 10 °C/min).

**Figure 6:**
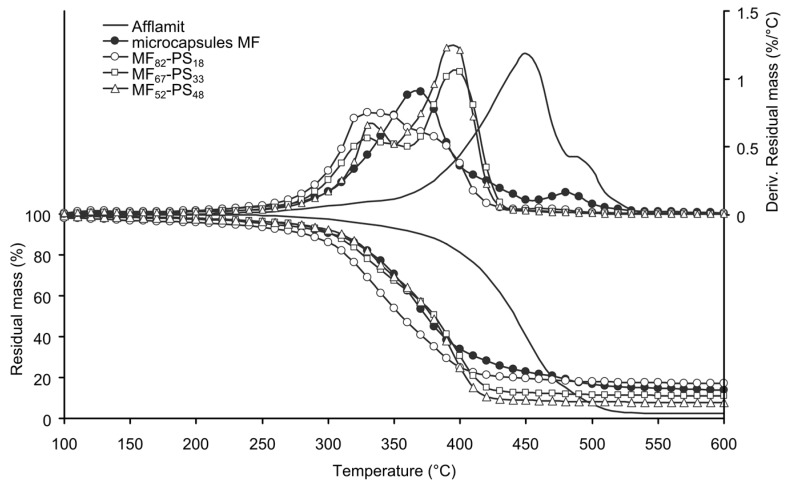
TG and DTG of RDP, MF microcapsules and PS-based microcapsules (N_2_, 10 °C/min).

Thermal stability of the microcapsules plays an important role to envisage their introduction into the nonwoven substrate and to confer flame retardant properties to it; therefore, it was studied by TGA. Figure 5 and Figure 6 illustrates the TGA thermograms of core solution, MF microcapsules and PMMA-based microcapsules or PS-based microcapsules, as well as their derivatives.

The thermal behaviour of RDP exhibits degradation in three steps. The first one occurs between 200 and 350 °C with 6% weight loss; the second, and main step, reflects 81% weight loss between 350 and 480 °C; and the last step reflects 11% weight loss between 480 and 550 °C. Finally, a RDP residue of 2% remains after total degradation. The volatilization of RDP in the second step is much lower than the degradation temperature of PET [39]. The presence of residue is related to the trans-esterification of P–O–C bond with phenolic group [43]. The thermal behaviour of the MF microcapsules occurs in two main stages, i.e., from 250–450 °C, and 450–550 °C. The first weight loss (76%) is not only attributed to the partial degradation of RDP, but also to the release of ammonia from the thermal degradation of the melamine derivatives. Above 500 °C, the thermal degradation of melamine takes place with the break-down of the three-dimensional polymer structure, leading to the formation of cyameluric structures, ammonia, and formaldehyde [27]. At the end of degradation, the stable residue is about 15%. This value is correlated to the amount of RDP present in the capsules and is in accordance with our previous study [38].

Thermal degradation of microcapsules based on PMMA occurs in four consecutive stages. The temperature at 5% of weight loss for all the PMMA based samples are close to each other, and can be attributed to the presence of solvent or GMA residues. The formation of the outer polymeric shell decreases the thermal stability of the microcapsules. Considering only the three last degradation stages, the temperature ranges and the weight loss are the same meaning that the samples have the same behaviour (Table 3). Additionally, it can be noticed that there is no correlation between the measured residue and the amount of PMMA. The thermal stability of the sample MF_52_-PMMA_48_ is lower than the two other samples due to the rapid growth of the polymeric shell around the particles and the formation of shorter macromolecular chains (Figure 5). From the data listed in Table 3, the formulation of the labelled sample MF_67_-PMMA_33_ allows to obtain the best thermal stability.

Degradation of PS based microcapsules occurs in two consecutive steps and the samples have a better thermal stability than the PMMA based ones (Figure 6). Furthermore, the temperature at 5% in weight loss increases from 225 to 273 or 277 °C when increasing the amount of PS from 18% to 33% or 48% (Table 3). It can also be observed that the increase of styrene monomers in the formulation bath provides a better thermal stability of the samples due to the formation of longer macromolecular chains. The maximum degradation temperature of the first stage tends to decrease with the increase of styrene, whereas for the second one increases. Furthermore, the weight loss of the samples during the first degradation stage decreases when increasing the PS amount, unlike what occurs during the second stage. Except for the first sample, the determined residue is in accordance with the amount of MF microcapsules in the sample.

### 3.3. Structures and Physical Properties of the Impregnated Nonwoven Fabrics

**Figure 7:**
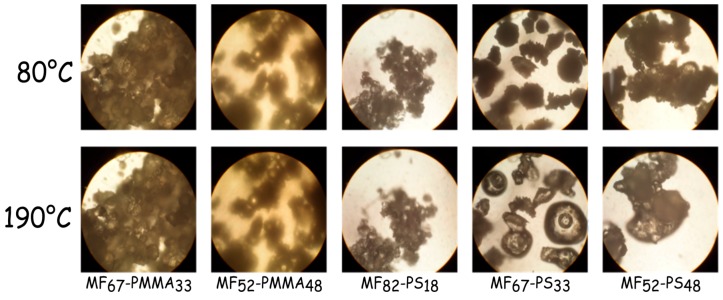
Optical micrographs of microcapsule samples heating with a microscope equipped with a heating plate (at 80 and 190 °C).

Before impregnation, the softening point of the microcapsules samples was determined with a Kofler bench (Table 4) and checked by optical microscopy equipped with a heating plate (Figure 7). The softening point of each sample is about the glass temperature of the outer polymeric shell. For the microcapsules based on PMMA, it increases from 98 to 110 °C with increasing the monomer amount in the formulation batch. For the samples based on PS, the amount of monomer does not change the softening temperature and it is closed to 80 °C. The Figure 7 shows the evolution of the microcapsule appearance with the temperature. From this figure, samples labelled MF_67_-PS_33_ and MF_58_-PS_42_ are sticky, nevertheless it the amount of outer shell is less than 33% the microcapsules are slightly tacky. Furthermore, for the microcapsules based PMMA, even at 190 °C, the adhesive properties are relatively low. Therefore, the formulation parameters, such as the kind of monomer used and the quantity, influence the adhesive properties of these microcapsules. In both cases, the determined softening point is sufficient to envisage the impregnation of the microcapsules onto nonwoven fabric.

**Figure 8:**
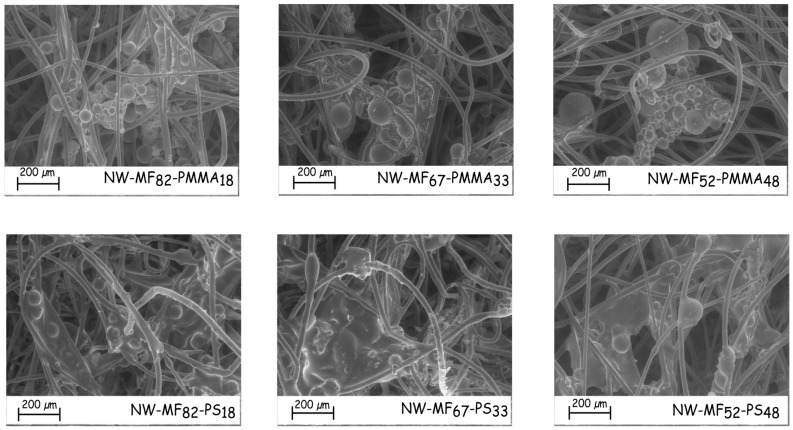
SEM micrographs of the impregnated nonwoven fabrics.

The amount of microcapsules impregnated in the nonwoven fabric increases with the percentage weight of the outer shell in the case of the microcapsules based on PS (Table 5). Most of these particles seem to be aggregated and dispersed into a polymeric film (Figure 8), which linked the fibres together. The film-forming ability of the PMMA outer shell does not occur in the same way. Microcapsules are less embedded in a film and are more located onto the surface fibre, even if most of them are aggregated to themselves (Figure 8). Surprisingly, the dry deposit weight of the sample impregnated with the microcapsules MF_67_-PMMA_33_ is higher than the others. In fact, the microcapsules are less aggregated and, therefore, may penetrate more in the nonwoven structure. Furthermore, the distribution of the particles in/on the nonwoven fabric is not homogenous, and they are more located on the surface than in the inner textile structure.

Air permeability and thickness of the nonwoven samples do not vary a lot according to the dry deposit weight and the type of microcapsules used. Nevertheless, the thickness of the treated sample slightly increases compared to raw sample (4.71 ± 0.09), and especially for the NW-MF_67_-PMMA_33_ sample. The air permeability decreases from 3090 L/m^2^/s for the non-related sample to about 2200 L/m^2^/s for the impregnated samples. The values are mainly influenced by the dry deposit weight for the samples. Thus, the functionalization of the nonwoven fabric from this process does not affect the physical properties of the textile, which maintains the structure of the fabric.

### 3.4. Flame Retardant Properties of the Impregnated Nonwoven Fabrics

**Figure 9:**
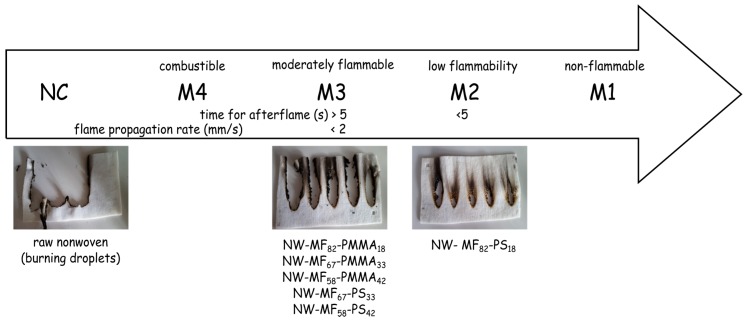
NF P 92-504 test results.

The flame retardant properties of the nonwoven samples were evaluated from the NF P92-504. This test was selected to conduct a comparative determination of the influence of the particles on the nonwoven substrate. The results reported in Figure 9 show that only one sample, the NW-MF_82_-PS_18_, can be classified as M2, and the others as M3. The main difference of these samples is related not only to the selected outer shell, but also to the loading content in RDP. Thus, for the same loading content and a higher dry deposit weight, the sample NW-MF_82_-PMMA_18_ (flame propagation rate of 1.0 ± 0.2 mm/s) is only M3, which is in concordance with the thermal stability determined by TGA. When the amount of the PMMA outer shell (NW-MF_58_-PMMA_42_) increases, the flame propagation increases, too, from 1.0 ± 0.2 to 1.5 ± 0.2 mm/s. The dry deposit weight affects also the flame propagation rate, since the sample NW-MF_82_-PMMA_18_ containing 18 g/m^2^ of capsules has a flame propagation rate value closed to the NW-MF_82_-PMMA_18_ (1.1 ± 0.3 mm/s). Furthermore, the two others nonwoven samples containing PS-based microcapsules have similar behaviour of NW-MF_58_-PMMA_42_; therefore, the flame retardant properties are mainly dependent on the kind of polymer entrapped by the MF microcapsules and the loading content of RDP.

## 4. Conclusions

In this work, resorcinol bis(diphenyl phosphate) has been successfully encapsulated by an in situ polymerization following by a suspension polymerization. The main aims of the formulation of a double-layered shell is to make it easier to handle of the flame-retardant compound and to allow its incorporation into a nonwoven PET substrate by a simple padding process without the use of a polymeric binder. In this study, the role of the outer shell is to link the microcapsules to the fibres with a heat treatment. The effect of the loading content on the thermal stability, textile properties, and flame retardant behaviour was studied. The thermal stability of the microcapsules depends on the choice of the polymer forming the outer shell and also the amount of monomer used in the formulation. The presence of microcapsules has a low impact on the air permeability and thickness of the nonwoven samples, even if they are more impregnated on the surface than in the inner structure. NF P92-504 test was used to evaluate the flame retardancy of the textile fabrics. A comparison of the samples shows that PS based microcapsules with a high loading content of RDP allows to reach the M2 classification, on the other side the remaining samples are M3. Some differences about the flame propagation rates were determined and mainly attributed to the loading content of RDP and the amount of microcapsules impregnated.

## Figures and Tables

**Table 1:** Composition of the double-layered shell microcapsules.

Sample label	Microcapsules MF		Outer shell
	RDP (wt %)	MF shell (wt %)	(wt %)
MF_82_-PMMA_18_	82		18% PMMA
	68	14	
MF_67_-PMMA_33_	67		33% PMMA
	55	12	
MF_52_-PMMA_48_	52		48% PMMA
	43	9	
MF_82_-PS_18_	82		18% PS
	68	14	
MF_67_-PS_33_	67		33% PS
	55	12	
MF_52_-PS_48_	52		48% PS
	43	9	

**Table 2:** Important peaks in the FTIR spectra and their assignments.

Wavenumbers (cm^−1^)				Assignments
Afflamit PLF 280	Microcapsules MF	Microcapsules MF_67_-PMMA_33_	Microcapsules MF_67_-PS_33_	
	3350	3350	3350	ν (–OH, –NH, –NH_2_)
3100–3000	3100–3000	3100–3000	3100–3000	ν (=C–H)
2970–2840	2970–2840	2970–2840	2970–2840	ν (–C–H)
		1720		ν (C=O)
1594, 1485	1594, 1485	1594, 1485	1625, 1594, 1485	ν (C=C)
	1555	1555	1555	ν (C=N)
		1440		δ (C–H)
			1359	δ (C–H)–CH_2_
1298, 964	1298, 964	1298, 964	1298, 964	ν (P=O)
		1200		ν (C–O–C)
	1200–1170	1200–1170	1200–1170	ν (C–N)
1195	1195	1195	1195	ν (Aro–O)
1073	1073	1073	1073	ν (P–O–(ph))
		887		δ (C–O)
	810	810	810	Triazine out of plane bend
			750–700	Ring deformation

**Table 3:** Thermal parameters obtained from TG and DTG curves of microcapsules samples.

Sample label	*T*_5%_	*T*_max_ (°C)/∆m (%)				Residue at 600 °C
	(°C)	I stage	II stage	III stage	IV stage	(%)
MF_82_-PMMA_18_	150	130/5	277/17	338/29	365/40	9
MF_67_-PMMA_33_	150	129/5	-	335/40	359/39	16
MF_52_-PMMA_48_	171	171/6	271/31	336/15	379/46	2
MF_82_-PS_18_	225	342/55	382/28	-	-	17
MF_67_-PS_33_	277	326/38	398/51	-	-	11
MF_52_-PS_48_	273	333/31	394/61	-	-	8

**Table 4:** Softening of the microcapsules.

Sample label	Softening point (°C)
MF_82_-PMMA_18_	98
MF_67_-PMMA_33_	106
MF_52_-PMMA_48_	110
MF_82_-PS_18_	81
MF_67_-PS_33_	81
MF_52_-PS_48_	83

**Table 5:** Physical properties of the impregnated nonwoven samples.

Sample label	Mass of dry deposit per unit area	Air permeability	Thickness
	(g/m^2^)	(L/m^2^/s)	(mm)
NW-MF_82_-PMMA_18_	13.9	2286 ± 35	4.74 ± 0.12
NW-MF_67_-PMMA_33_	18.0	2113 ± 140	4.92 ± 0.08
NW-MF_52_-PMMA_48_	13.8	2214 ± 170	4.64 ± 0.17
NW-MF_82_-PS_18_	13.5	2470 ± 110	4.74 ± 0.14
NW-MF_67_-PS_33_	14.9	2236 ± 125	4.65 ± 0.15
NW-MF_52_-PS_48_	16.7	2113 ± 104	4.74 ± 0.13

## Abbreviations

The following abbreviations are used in this manuscript:

FR	flame retardant
FTIR	Fourier transform infrared spectroscopy
GMA	glycidyl methacrylate
MF	melamine-formaldehyde resin
NW	nonwoven
PET	poly(ethylene terephtalate)
PMMA	poly(methyl methacrylate)
PS	polystyrene
RDP	resorcinol bis(diphenyl phosphate)
SEM	scanning electron microscopy
